# Well-posed continuum equations for granular flow with compressibility and *μ*(*I*)-rheology

**DOI:** 10.1098/rspa.2016.0846

**Published:** 2017-05-03

**Authors:** T. Barker, D. G. Schaeffer, M. Shearer, J. M. N. T. Gray

**Affiliations:** 1 School of Mathematics and Manchester Centre for Nonlinear Dynamics, University of Manchester, Oxford Road, Manchester M13 9PL, UK; 2 Mathematics Department, Duke University, Box 90320, Durham, NC 27708-0320, USA; 3 Department of Mathematics, North Carolina State University, Raleigh, NC 27695-8205, USA

**Keywords:** granular flow, continuum modelling, rheology

## Abstract

Continuum modelling of granular flow has been plagued with the issue of ill-posed dynamic equations for a long time. Equations for incompressible, two-dimensional flow based on the Coulomb friction law are ill-posed regardless of the deformation, whereas the rate-dependent *μ*(*I*)-rheology is ill-posed when the non-dimensional inertial number *I* is too high or too low. Here, incorporating ideas from critical-state soil mechanics, we derive conditions for well-posedness of partial differential equations that combine compressibility with *I*-dependent rheology. When the *I*-dependence comes from a specific friction coefficient *μ*(*I*), our results show that, with compressibility, the equations are well-posed for all deformation rates provided that *μ*(*I*) satisfies certain minimal, physically natural, inequalities.

## 1. Introduction

Much effort has been devoted to formulating constitutive laws for continuum models of granular materials [1–5]. However, the lack of acceptable dynamic theories, i.e. well-posed equations in the sense of Joseph & Saut [6], for granular flow has severely hampered progress in modelling many geophysical and industrial problems. In the simplest class of models, flow is described by partial differential equations (PDEs) for the density, the velocity vector and the stress tensor; conceptually, such models are hardly more complicated than the Navier–Stokes equations. The equations represent conservation laws for mass and momentum coupled to constitutive equations to close the system. However, despite the appeal of their simplicity, they have been plagued with ill-posedness, i.e. small perturbations grow at an unbounded rate in the limit that their wavelength tends to zero [6]. Such behaviour is clearly unphysical. However, the immediate practical implication of ill-posedness is that numerical computations either blow-up, even at finite resolution, or do not converge to a well-defined solution as the grid is refined, i.e. the numerical results are grid dependent [7–10].

The first model of this type [2,11,12] specifies constitutive laws that represent a tensorial generalization of the work of de Coulomb [13] on earthwork fortifications. In the language of plasticity theory, it is a rate-independent, rigid/perfectly plastic model with a yield condition based on friction between the grains. However, it was shown to be ill-posed in all two-dimensional contexts and all realistic three-dimensional contexts [2]. Critical-state soil mechanics (CSSM) [1] is a sophisticated elaboration of Coulomb behaviour that allows for compressibility. It also suffers from ill-posedness, depending on the degree of consolidation. This ill-posedness is much less severe than for a Coulomb material [3,12], but is still physically unsatisfactory and introduces potential issues for the numerical simulation of transient granular flows. More recently, the *μ*(*I*)-rheology [4,5,14] introduces a modest amount of rate dependence into (incompressible) Coulomb behaviour through the non-dimensional *inertial number*, which is proportional to the shear rate and inversely proportional to the square root of the pressure. As shown in Barker *et al.* [9], this theory leads to well-posed (two dimensional) equations in a significant region of state space, but it is ill-posed at both low and high inertial numbers.

This paper presents an analysis of constitutive equations that extend the incompressible *μ*(*I*)-rheology of Jop *et al.* [5] to compressible deformations, through combination with CSSM. The main result is that, in two dimensions, compressible *I*-dependent equations can be made well-posed for all densities, for all stress states and for all deformation rates. In other words, to obtain well-posedness, Coulomb behaviour is modified by including only two natural, fairly small, perturbations of the theory, namely compressibility and rate dependence. Following this very general treatment, which has implications for many existing formulations [15–17], we elucidate our findings with an illustrative model that includes physically motivated features and reduces to the *μ*(*I*)-rheology in the incompressible limit. This has the advantage that it retains the conceptual simplicity of the original theory. Although we consider only two-dimensional flow, it should be noted that in numerous cases it has been found that flow in two dimensions is more prone to ill-posedness than in three [2,3,18]. Thus, we anticipate that the corresponding three-dimensional equations including these effects will also be well-posed.

Currently, a wide range of new constitutive laws for granular materials are being developed including the *μ*(*I*)-rheology [4,5], elasto-plastic formulations [19,20], non-local rheologies [21–24], kinetic theory [25], as well as Cosserat [26], micro-structural [27] and hypoplastic theories [28]. Enormous progress has been made over the past decade and there is the realistic and exciting prospect that practical granular flows, which span the solid-like, liquid-like and gaseous regimes, may shortly be described by continuum models. In this paper, we seek to understand one of the conceptually simplest formulations that leads to well-posed equations.

In §2, we introduce the equations to be studied and formulate our well-posedness result for them. This theorem is proved in §§3 and 4. In §5, we solve the new equations for steady, uniform chute flow. In two appendices, we summarize key ideas from CSSM and survey topics regarding ill-posed partial differential equations.

## 2. Governing equations

Dense granular flow is described by the solids volume fraction *ϕ*, the velocity vector ***u*** and the stress tensor ***σ***. In two dimensions, this constitutes six scalar unknowns that are spatially and temporally dependent. These are governed by conservation laws plus constitutive relations. Conservation of mass gives the scalar equation

2.1 $$(\partial_{t}+u_{j}\partial_{j})\phi +\phi \;\text{div}\;u=0,$$

and conservation of momentum gives the vector equation

2.2 $$\rho_{*}\phi (\partial_{t}+u_{j}\partial_{j})u_{i}=\partial_{j}\sigma_{ij}+\rho_{*}\phi g_{i},$$

where ρ_*_ is the constant intrinsic grain density and *g* is the acceleration due to gravity. Closure of these equations is achieved through three constitutive relations.

### (a) The Coulomb constitutive model

For a Coulomb material, which is assumed to be incompressible, the first constitutive relation states that *ϕ* is a constant. This then reduces (2.1) to the

2.3 Flow rule: div u=0.

For the next constitutive relation, the stress tensor

2.4 $$\sigma_{ij}=-p\delta_{ij}+\tau_{ij}$$

is decomposed into a pressure term (where *p*=−*σ*_*ii*_/2) plus a trace-free tensor ***τ*** called the deviatoric stress. The second relation is then the

2.5 Yield condition: ∥τ∥=μp,

where *μ* is a constant and for any tensor ***T*** the norm is defined by

2.6 $$∥T∥=\sqrt{\frac{T_{ij}T_{ij}}{2}}.$$

This yield condition expresses the idea that a granular material cannot deform unless the shear stress is sufficient to overcome friction.^1^ The third constitutive relation requires that the eigenvectors of the deviatoric stress tensor and the deviatoric strain-rate tensor^2^

**Figure 1.**
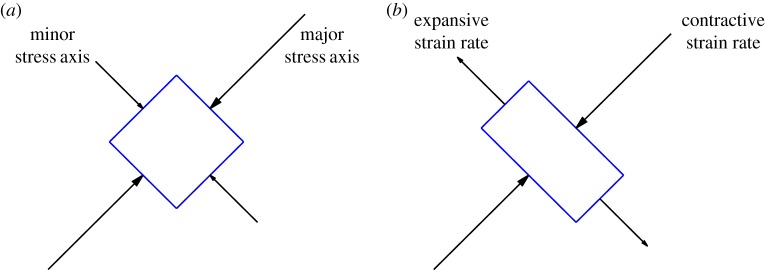
(*a*) Illustrative stress eigenvectors; along the major axis the stress eigenvalue is −(*p*+∥***τ***∥), with the minus sign indicating compression. (*b*) A possible material deformation that is consistent with the stress field in (*a*). (Online version in colour.)

2.7 $$D_{ij}=\frac{1}{2}(\partial_{j}u_{i}+\partial_{i}u_{j})-\frac{1}{2}(\text{div}\;u)\delta_{ij}$$

are aligned (see figure 1 for motivation), which may be written

2.8 $$\text{Alignment:}\;\frac{D_{ij}}{∥D∥}=\frac{\tau_{ij}}{∥\tau ∥}.$$

In words, the above equation may be interpreted as asserting that in the space of trace-free symmetric 2×2 matrices, *which is two-dimensional*, ***D*** and ***τ*** are parallel. Thus, this matrix equation entails only one scalar relation. For reference below we record that

2.9 $$D=\frac{1}{2}\left[\begin{array}{cc} \partial_{1}u_{1}-\partial_{2}u_{2} & \partial_{1}u_{2}+\partial_{2}u_{1}\\ \partial_{1}u_{2}+\partial_{2}u_{1} & \partial_{2}u_{2}-\partial_{1}u_{1} \end{array}\right].$$

It is customary [2,5] to process these equations by expressing the deviatoric stress ***τ*** in terms of *p* and the strain rate as follows:

2.10 $$\tau_{ij}=∥\tau ∥\frac{\tau_{ij}}{∥\tau ∥}=\mu p\frac{D_{ij}}{∥D∥},$$

where we have invoked (2.5) and (2.8). We may substitute (2.10) into (2.2) to obtain

2.11 $$\rho_{*}\phi (\partial_{t}+u_{j}\partial_{j})u_{i}=\partial_{j}\left[\frac{\mu p}{∥D∥}D_{ij}\right]-\partial_{i}p+\rho_{*}\phi g_{i}$$

and the resulting equation, together with (2.3), gives three equations for pressure *p* and velocity ***u***. In form, at least, these equations resemble the incompressible Navier–Stokes equation. However, in two dimensions (as considered here) they are always ill-posed [2].

### (b) Incompressible *μ*(*I*)-rheology

Work described by the Groupement De Recherche Milieux Divisés [4] has significantly improved the Coulomb model by including some rate dependence (in the sense of plasticity [29]) in the yield condition while making no changes in the incompressible flow rule (2.3) and the alignment condition (2.8). Specifically, a wide range of experiments are captured by replacing the constant *μ* in (2.5) by an increasing function *μ*(*I*) of the *inertial number*,

2.12 $$I=\frac{2d∥D∥}{\sqrt{p/\rho_{*}}},$$

where *d* is the particle diameter. The expression

2.13 $$\mu (I)=\mu_{1}+\frac{\mu_{2}-\mu_{1}}{I_{0}/I+1},$$

where *μ*_1_, *μ*_2_ and *I*_0_ are constants with *μ*_2_>*μ*_1_, is a frequently used form [30]. Below we shall assume that

2.14 $$\mu^{\prime }(I)>0\;\text{and}\;\mu^{\prime \prime }(I)<0.$$

The modified yield condition changes (2.11) to read

2.15 $$\rho_{*}\phi (\partial_{t}+u_{j}\partial_{j})u_{i}=\partial_{j}\left[\frac{\mu (I)p}{∥D∥}D_{ij}\right]-\partial_{i}p+\rho_{*}\phi g_{i}.$$

The effect of this seemingly small perturbation is profound. Unlike for Coulomb material, equations (2.15) and (2.3) are linearly well-posed for *I*, *μ* satisfying

2.16 $$4\nu^{2}-4\nu +\mu^{2}{\left(1-\frac{\nu }{2}\right)}^{2}\leq 0,\;\text{where}\;\nu =\frac{I}{\mu }\frac{\text{d}\mu }{\text{d}I}.$$

For the specific *μ*(*I*)-curve (2.13) this inequality covers a significant range of inertial numbers, specifically when the deformation rate is neither too small nor too large relative to the pressure. Outside of this range, the maximal-order linear stability analysis and numerical simulations show that perturbations grow exponentially with growth rates tending to infinity as their wavelength is reduced [9]. This behaviour is the hallmark of ill-posedness and leads to unphysical numerical solutions that strongly depend on the grid resolution used.

### (c) Compressibility and *I*-dependent rheology

We refer to CSSM (see appendix A) for guidance in introducing compressibility into the rheology. Thus, we make no change in the alignment condition (2.8); we assume *ϕ*-dependence in the yield condition,

2.17 ∥τ∥=Y(p,ϕ,I),

and we allow for volumetric changes by introducing a new function *f*(*p*,*ϕ*,*I*) and modifying the flow rule to

2.18 div u=2f(p,ϕ,I)∥D∥.

To get well-posed equations, our analysis (see §3) shows that the yield condition and the flow-rule functions must be related by the equation^3^

2.19 $$\frac{\partial Y}{\partial p}-\frac{I}{2p}\frac{\partial Y}{\partial I}=f+I\frac{\partial f}{\partial I}$$

and satisfy the inequalities

2.20*a* $$\partial_{I}Y>0$$

and

2.20*b* $$\partial_{p}f-\frac{I}{2p}\partial_{I}f<0.$$

We may now state our main result, the well-posedness theorem for the system (2.1), (2.2), (2.8), (2.17), (2.18), which we call the CIDR equations. (Mnemonic: compressible *I*-dependent rheology.)

**Theorem 2.1**

Under hypotheses (2.19) and (2.20), the CIDR system is linearly well-posed.

The term *linearly well-posed* is defined in appendix B, and the result is proved in §§3 and 4. Table 1 contains a comparison between the conditions for linear well-posedness for the different constitutive models that have been discussed here.

**Table 1.** Table of criteria for linear well-posedness.

**model**	Coulomb	incomp. *μ*(*I*)	CSSM	CIDR
**conditions**	always ill-posed	(2.16)	always ill-posed	(2.19), (2.20)

**Remark 2.2**

The *I*-dependence in these equations need not relate to a friction coefficient *μ*(*I*). In §2e, we connect the equations to *μ*(*I*)-rheology.

### (d) Derivation of evolution equations

To place the equations in a larger continuum-mechanics context, we show that the CIDR equations of motion can be rewritten as a system of three evolution equations for the velocity ***u*** and the solids fraction *ϕ*. In form, these equations are analogous to the Navier–Stokes equations for a viscous, compressible fluid. We make no use of this form of the equations in our proof of well-posedness.

We want to eliminate stresses from the equations of motion. To this end, we propose to solve for the mean stress *p* using the flow rule (2.18), which we rewrite as

2.21 $$f(p,\phi ,I)=\frac{\text{div}\;u}{2∥D∥}.$$

Note that *f*(*p*,*ϕ*,*I*) depends on *p* both directly in its first argument and indirectly through $I=2d∥D∥/\sqrt{p/\rho_{*}}$ in its third argument. However,

2.22 $$\frac{\partial }{\partial p}\left[f\left(p,\phi ,\frac{2d∥D∥}{\sqrt{p/\rho_{*}}}\right)\right]=\partial_{p}f-\frac{I}{2p}\partial_{I}f,$$

which by assumption (2.20b) is non-zero. Thus, we may apply the implicit function theorem to (2.21) to solve *p*=*P*(∇***u***,*ϕ*).^4^ Given this, we may define

$$T(\nabla u,\phi )=Y(P(\nabla u,\phi ),\phi ,I(\nabla u,\phi )),\;\text{where}\;I(\nabla u,\phi )=\frac{2d∥D∥}{\sqrt{P(\nabla u,\phi )/\rho_{*}}}$$

and substitute into conservation of momentum to obtain an equation

2.23 $$\rho_{*}\phi (\partial_{t}+u_{j}\partial_{j})u_{i}=\partial_{j}\left[\frac{T(\nabla u,\phi )}{∥D∥}D_{ij}\right]-\partial_{i}[P(\nabla u,\phi )]+\rho_{*}\phi g_{i}.$$

This equation, along with (2.1), gives a system of three evolution equations for the velocity ***u*** and the solids fraction *ϕ*. It is possible that previous formulations of compressible *μ*(*I*) equations [15–17] may be seen as CIDR equations with specific constitutive laws specified. In this paper, we choose to elucidate the well-posedness result with more generic choices of *f* and *Y* in order to remain impartial.

### (e) Connection to *μ*(*I*)-rheology

Without making any attempt to be general, we illustrate one example of how *μ*(*I*)-rheology may be included in constitutive relations of the form (2.17), (2.18). Motivated by equation (.3) in appendix A, we make the ansatz

2.24*a* $$Y(p,\phi ,I)=\alpha (I)p-\frac{p^{2}}{C(\phi )}$$

and

2.24*b* $$f(p,\phi ,I)=\beta (I)-\frac{2p}{C(\phi )}.$$

**Figure 2.**
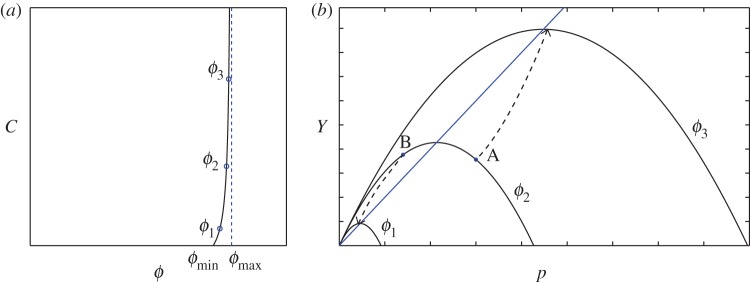
(*a*) An example curve for the function *C*(*ϕ*) with a minimum solids volume fraction *ϕ*_min_ and a vertical asymptote at *ϕ*_max_. (*b*) Nested yield surfaces of the form (2.24) for a fixed value of *I* with differing solids volume fractions. (The solid blue line, the dashed arrows and the labels A and B refer to a discussion of CSSM in appendix A.)

In these equations, it is worth emphasizing that *p*,*ϕ*,*I* are treated as independent variables, not to be confused with the dependence of *I* on *p* in the previous subsection. The function *C*(*ϕ*) is an increasing function of *ϕ*. As *ϕ* varies (with *I* fixed) the yield loci ∥***τ***∥=*Y* (*p*,*ϕ*,*I*) derived from (2.24a) form a nested family of convex curves in stress space (figure 2*b*). Observe from (2.18) that deformation without volumetric strain is possible if *f*(*p*,*ϕ*,*I*)=0; i.e. for (2.24b), if *p*/*C*(*ϕ*)=*β*(*I*)/2. Substituting this formula into (2.17) and using (2.24a), we derive

$$∥\tau ∥=\left[\alpha (I)-\frac{\beta (I)}{2}\right]p,$$

for such isochoric deformation to be possible. Thus, to recover the yield condition ∥***τ***∥=*μ*(*I*)*p* of the *μ*(*I*)-rheology, let us require that

2.25 $$\alpha (I)-\frac{\beta (I)}{2}=\mu (I).$$

**Lemma 2.3**

*Equations (2.19) and (2.25) imply that*

2.26 $$\alpha (I)=\frac{4}{5}\mu (I)+\frac{12}{25}I^{-2/5}\int_{0}^{I}J^{-3/5}\mu (J)\;dJ$$

*and*

2.27 $$\beta (I)=-\frac{2}{5}\mu (I)+\frac{24}{25}I^{-2/5}\int_{0}^{I}J^{-3/5}\mu (J)\;dJ.$$

**Proof.**

Substituting the relations (2.24) into (2.19), and using (2.25) to eliminate *β*, we derive the linear ordinary differential equation for *α*=*α*(*I*):

2.28 $$\frac{5}{2}I\alpha^{\prime }(I)+\alpha (I)=2\mu (I)+2I\mu^{\prime }(I).$$

Solving this linear equation for *α*(*I*), with an integrating factor, we obtain

$$I^{2/5}\alpha (I)=\frac{4}{5}\int_{0}^{I}J^{-3/5}\mu (J)\;dJ+\frac{4}{5}\int_{0}^{I}J^{2/5}\mu^{\prime }(J)\;dJ,$$

from which the formula (2.26) follows after integrating the second integral by parts. Finally, substituting this formula for *α*(*I*) into (2.25), we obtain the formula (2.27) for *β*(*I*). ■

**Lemma 2.4**

The yield condition and flow-rule function (2.24*a*,*b*) that follow from (2.26), (2.27) verify hypotheses (2.19) and (2.20), provided *μ*(*I*) satisfies (2.14).

**Proof.**

Of course (2.19) is satisfied because this equation was imposed in deriving (2.26), (2.27).

Differentiating (2.24b), we see that ∂_*p*_*f*(*p*,*ϕ*,*I*)=−2/*C*(*ϕ*)<0. To calculate ∂_*I*_*f*(*p*,*ϕ*,*I*), we first reparametrize the integral in (2.27) to obtain $\beta (I)=-\frac{2}{5}\mu (I)+\frac{24}{25}\int_{0}^{1}s^{-3/5}\mu (sI)\;ds$. Then

$$\partial_{I}f(p,\phi ,I)=\beta^{\prime }(I)=-\frac{2}{5}\mu^{\prime }(I)+\frac{24}{25}\int_{0}^{1}s^{2/5}\mu^{\prime }(sI)\;ds.$$

By (2.14), *μ*′′(*I*)<0, so *μ*′(*sI*)>*μ*′(*I*) for 0<*s*<1. Thus,

$$\beta^{\prime }(I)>\mu^{\prime }(I)\left\{-\frac{2}{5}+\frac{24}{25}\int_{0}^{1}s^{2/5}\;ds\right\}=\mu^{\prime }(I)\left\{\frac{24}{35}-\frac{2}{5}\right\}>0,$$

the last inequality using (2.14). Consequently,

2.29 $$\partial_{p}f(p,\phi ,I)-\frac{I}{2p}\partial_{I}f(p,\phi ,I)<0,$$

proving inequality (2.20b).

For inequality (2.20a), we reparametrize the integral (2.26) and differentiate to obtain

$$\partial_{I}Y(p,\phi ,I)=\alpha^{\prime }(I)p=p\left(\frac{4}{5}\mu^{\prime }(I)+\frac{12}{25}\int_{0}^{1}s^{2/5}\mu^{\prime }(sI)\;ds\right)>0,$$

as desired. ■

### (f) The incompressible limit

Based on an analogy with CSSM, let us suppose that *C*(*ϕ*) is a sensitive function of *ϕ*, say of the form

2.30 $$C(\phi )=\frac{\rho_{⋆}gd}{b}\overset{ˇ}{C}\left(\frac{\phi -\phi_{\min}}{\phi_{\max}-\phi_{\min}}\right),$$

where *g* is the constant of gravitational acceleration, *b* is a non-dimensional parameter and the factor *ρ*_⋆_*gd* gives *C* the dimensions of pressure. The non-dimensional function $\overset{ˇ}{C}$ has an argument that is dependent on the minimum solids fraction *ϕ*_min_ for sustained stress transmission between grains (random loose packing^5^) and *ϕ*_max_, which is the maximum packing fraction that can be attained. Typically, *Δϕ*=*ϕ*_max_−*ϕ*_min_ is small. For definiteness we may take

2.31 $$\overset{ˇ}{C}(y)=\frac{y}{1-y},$$

as in figure 2. Note that *C*(*ϕ*) diverges as *ϕ*→*ϕ*_max_; thus, (2.30) requires that *ϕ* is confined to a narrow range,

2.32 $$\phi_{\min}\leq \phi <\phi_{\max}.$$

In physical terms, the maximum solids fraction *ϕ*_max_ represents the jamming threshold. We call the limit *Δϕ*→0 *incompressible* because, as may be seen from (2.32), the density of the material becomes essentially constant.

**Lemma 2.5**

As *Δϕ*→0, the CIDR equations reduce to the equations of incompressible *μ*(*I*)-rheology, (2.3), (2.15).

**Proof.**

We process the CIDR equations, which have the six unknowns *ϕ*, *u*_*i*_ and *σ*_*ij*_, as follows. First, we reduce to five unknowns—*ϕ*, *u*_*i*_, *p* and *τ*=∥***τ***∥—by recalling the definition (2.4) and the alignment condition (2.8) to write

$$\sigma_{ij}=-p\delta_{ij}+\tau \frac{D_{ij}}{∥D∥}.$$

Next we use the yield condition to eliminate *ϕ*, reducing this number to four. Specifically, substituting (2.24a) into (2.17), we write the yield condition

2.33 $$\tau =\alpha (I)p-\frac{p^{2}}{C(\phi )}.$$

Solving (2.33) for *ϕ* we obtain

2.34 $$\phi =\Phi (\nabla u,p,\tau )=C^{-1}\left(\frac{p^{2}}{\alpha (I)p-\tau }\right),$$

where the dependence on ∇***u*** comes from the fact that $I=2d∥D∥/\sqrt{p/\rho_{*}}.$ Substitution of this formula into the conservation laws (2.1), (2.2) yields the equations

2.35*a* $$(\partial_{t}+u_{j}\partial_{j})\Phi (\nabla u,p,\tau )+\Phi (\nabla u,p,\tau )\;\text{div}\;u=0$$

and

2.35*b* $$\rho_{*}\Phi (\nabla u,p,\tau )(\partial_{t}+u_{j}\partial_{j})u_{i}=\partial_{j}\left[\frac{\tau }{∥D∥}D_{ij}\right]-\partial_{i}p+\rho_{*}\Phi g_{i}.$$

Finally, we show that the flow rule (2.18) may be rewritten

2.36 $$\text{div}\;u=4\left[\frac{\tau }{p}-\mu (I)\right]∥D∥.$$

To see this, we combine (2.24b) with (2.25) to conclude

$$f(p,\phi ,I)=\beta (I)-\frac{2p}{C(\phi )}=2[\alpha (I)-\mu (I)]-\frac{2p}{C(\phi )}$$

and then substitute the relation *α*(*I*)=*τ*/*p*+*p*/*C*(*ϕ*) derived by manipulating (2.33). Thus, the system (2.35), (2.36) governs the evolution of the four unknowns *u*_*i*_, *p* and *τ*.

Now we claim that if *C*(*ϕ*) has the form (2.30), then (2.35), (2.36) is a singular perturbation of (2.3), (2.15). It follows from (2.30) that (2.34) has the expansion

2.37 $$\phi =\phi_{0}+\Delta \phi \overset{ˇ}{\Phi }(\nabla u,p,\tau ),\;\text{where}\;\overset{ˇ}{\Phi }(\nabla u,p,\tau )={\overset{ˇ}{C}}^{-1}\left(\frac{bp^{2}}{\rho_{*}gd[\alpha (I)p-\tau ]}\right).$$

Substituting (2.37) into the continuity equation (2.35a), we find

$$\Delta \phi (\partial_{t}+u_{j}\partial_{j})\overset{ˇ}{\Phi }(\nabla u,p,\tau )+[\phi_{0}+\Delta \phi \overset{ˇ}{\Phi }(\nabla u,p,\tau )]\;\text{div}\;u=0.$$

If *Δϕ*=0, then this equation reduces to div ***u***=0, although this is of course a highly singular limit. Thus, if *Δϕ*=0, the left-hand side of (2.36) vanishes, so this equation simplifies to the yield condition *τ*=*μ*(*I*)*p*, and substitution into (2.35b) yields (2.15). This proves the lemma. ■

## 3. Proofs, part I: linearization

### (a) An alternative formulation of the alignment condition

It is convenient to study the linearized equations with a reformulated alignment condition that describes stress in terms of eigenvectors of, rather than entries of, the stress tensor. Since ***τ*** defined by (2.4) has trace zero, it has eigenvalues^6^ ±∥***τ***∥. Taking *ψ* as the angle that the eigenvector with eigenvalue −∥***τ***∥ makes with the *x*_1_-axis gives

3.1 $$\tau =-∥\tau ∥\left[\begin{array}{cc} \cos2\psi & \sin2\psi \\ \sin2\psi & -\cos2\psi \end{array}\right],$$

which may be verified by checking that (cos⁡ψ, sin⁡ψ) is an eigenvector of this matrix with eigenvalue −∥***τ***∥. Thus, the stress tensor *σ*_*ij*_ is completely specified by the three scalars *p*, ∥***τ***∥ and *ψ*.

Focusing on the first rows of the strain-rate tensor (2.9) and of (3.1), we extract from the matrix equation (2.8) the vector equation

3.2 $$(\partial_{1}u_{1}-\partial_{2}u_{2},\partial_{1}u_{2}+\partial_{2}u_{1})=k(\cos2\psi ,\sin2\psi ),$$

where *k*=−2∥***D***∥<0. Since ***D*** and ***τ*** lie in the two-dimensional space of trace-free, symmetric matrices, (3.2) is equivalent to (2.8). It follows from (3.2) that

3.3 $$\text{Alt. alignment:}\;(\partial_{1}u_{2}+\partial_{2}u_{1})\cos2\psi -(\partial_{1}u_{1}-\partial_{2}u_{2})\sin2\psi =0.$$

In point of fact, this equation is slightly weaker than the alignment condition since (3.3) is consistent with the possibility that *k*>0 in (3.2); to rule out the latter possibility we impose the supplemental inequality^7^ that

3.4 $$(\partial_{1}u_{1}-\partial_{2}u_{2})\cos2\psi \leq 0.$$

### (b) The calculation

Substitution of the stress tensor (3.1) into the momentum balance equations (2.2) allows for the full set of equations to be written as

3.5*a* ρ∗ϕ(∂t+u1∂1+u2∂2)u1+∂1[p+τ cos⁡(2ψ)]+∂2[τ sin⁡(2ψ)]=ρ∗ϕg1,

3.5*b* ρ∗ϕ(∂t+u1∂1+u2∂2)u2+∂1[τ sin⁡(2ψ)]+∂2[p−τ cos⁡(2ψ)]=ρ∗ϕg2,

3.5*c* $$\begin{array}{rl} & (\partial_{t}+u_{1}\partial_{1}+u_{2}\partial_{2})\phi +\phi (\partial_{1}u_{1}+\partial_{2}u_{2})=0, \end{array}$$

3.5*d* $$\begin{array}{rl} & \partial_{1}u_{1}+\partial_{2}u_{2}=2f∥D∥ \end{array}$$

3.5*e* $$\begin{array}{rl} \text{and}\; & (\partial_{2}u_{1}+\partial_{1}u_{2})\cos(2\psi )+(\partial_{2}u_{2}-\partial_{1}u_{1})\sin(2\psi )=0. \end{array}$$

This system has five scalar unknowns, ***U***=(*u*_1_,*u*_2_,*ϕ*,*p*,*ψ*). In (3.5a), (3.5b), *τ* is a mnemonically suggestive abbreviation for the yield function *Y* (*p*,*ϕ*,*I*) in (2.17), and in (3.5d), a repetition of (2.18), the function *f* depends on arguments (*p*,*ϕ*,*I*) that are not written explicitly.

As in appendix B, to linearize the equations we substitute a perturbation of a base solution $U^{\left(0\right)}(x,t)$, say

3.6 $$U=U^{\left(0\right)}+\hat{U},$$

into the equations, retain only terms that are linear in the perturbation $\hat{U}$ and freeze the coefficients at an arbitrary point $\left(x^{*},t^{*}\right)$. It is convenient to temporarily drop most terms not of maximal order and estimate their effect in a calculation at the end of the argument. For example, this construction applied to (3.5c) yields the constant-coefficient, linear equation

3.7 $$(\partial_{t}+u_{1}^{*}\partial_{1}+u_{2}^{*}\partial_{2})\hat{\phi }+\phi^{*}(\partial_{1}{\hat{u}}_{1}+\partial_{2}{\hat{u}}_{2})=0,$$

where $u_{j}^{*}=u_{j}^{\left(0\right)}(x^{*},t^{*})$ and $\phi^{*}=\phi^{\left(0\right)}(x^{*},t^{*})$. Lower-order terms $\partial_{j}\phi^{*}\;\hat{u_{j}}$ and $\partial_{j}u_{j}^{*}\;\hat{\phi }$ in the full linearization of (3.5c) have been dropped in (3.7).

In expanding the fully nonlinear factor ∥***D***∥ in (3.5d), we may take advantage of the rotational invariance of the equations to arrange that ψ*=0; i.e. we may calculate in a rotated coordinate system for which, at $\left(x^{*},t^{*}\right)$, the *x*_1_-axis is the maximal stress axis. Then by the alignment condition (3.3), the base-state deviatoric strain-rate tensor is diagonal at $\left(x^{*},t^{*}\right)$

**Figure 3.**
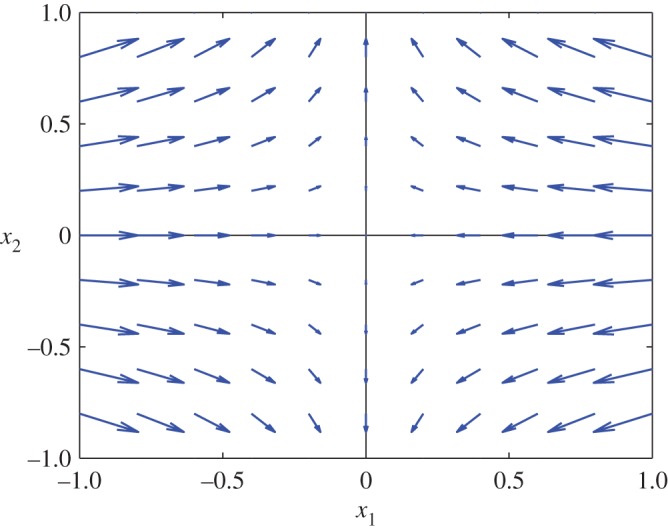
An example of a base-state velocity field for the strain-rate tensor (3.8) with $\partial_{1}u_{1}^{\left(0\right)}\equiv -1$ and $\partial_{2}u_{2}^{\left(0\right)}\equiv 1/2$. (Online version in colour.)

3.8 $$D^{*}=\left[\begin{array}{cc} \frac{\left(\partial_{1}u_{1}^{*}-\partial_{2}u_{2}^{*}\right)}{2} & 0\\ 0 & \frac{\left(\partial_{2}u_{2}^{*}-\partial_{1}u_{1}^{*}\right)}{2} \end{array}\right],$$

and by (3.4), in the 1,1-position of this matrix, $\partial_{1}u_{1}^{*}-\partial_{2}u_{2}^{*}<0$. This corresponds to non-zero compression along the major stress axis, as illustrated in figure 3. Now

3.9*a* $$∥(D^{*}+\hat{D})∥=\frac{1}{2}{\left[{\left(\partial_{1}u_{1}^{*}-\partial_{2}u_{2}^{*}+\partial_{1}{\hat{u}}_{1}-\partial_{2}{\hat{u}}_{2}\right)}^{2}+{\left(\partial_{2}{\hat{u}}_{1}+\partial_{1}{\hat{u}}_{2}\right)}^{2}\right]}^{1/2}$$

and

3.9*b* $$\approx ∥D^{*}∥-\frac{\left(\partial_{1}{\hat{u}}_{1}-\partial_{2}{\hat{u}}_{2}\right)}{2},$$

where the approximation follows from the expansion

$$\sqrt{{\left(-A+X\right)}^{2}+Y^{2}}=A-X+O(X^{2}+Y^{2}),$$

if *A*>0 and |*X*|,|*Y* |≪*A*. Thus, as given in table 2, the (local) linearization of ∥***D***∥ equals $-(\partial_{1}{\hat{u}}_{1}-\partial_{2}{\hat{u}}_{2})/2$.

**Table 2.** List of maximal-order linearizations of terms in (3.5a)–(3.5e), to assist in deriving (3.10a)–(3.10e). ${\hat{D}}_{11}=(\partial_{1}{\hat{u}}_{1}-\partial_{2}{\hat{u}}_{2})/2$ (only in this table).

term in (3.5a)–(3.5e)	contribution to (3.10a)–(3.10e)
∥***D***∥	$-{\hat{D}}_{11}$
*I*	$-\frac{I^{*}}{∥D^{*}∥}{\hat{D}}_{11}-\frac{I^{*}}{2p^{*}}\hat{p}$
∂j[τ cos⁡(2ψ)]	${\left(\partial_{p}\tau \right)}^{*}\partial_{j}\hat{p}+{\left(\partial_{\phi }\tau \right)}^{*}\partial_{j}\hat{\phi }$
	$\;+\;{\left(\partial_{I}\tau \right)}^{*}\left\{-\frac{I^{*}}{∥D^{*}∥}\partial_{j}{\hat{D}}_{11}-\frac{I^{*}}{2p^{*}}\partial_{j}\hat{p}\right\}$
∂j[τ sin⁡(2ψ)]	$2\tau^{*}\partial_{j}\hat{\psi }$
*f*∥***D***∥	$-f^{*}{\hat{D}}_{11}+∥D^{*}∥{\left(\partial_{p}f\right)}^{*}\hat{p}+∥D^{*}∥{\left(\partial_{\phi }f\right)}^{*}\hat{\phi }$
	$\;+\;∥D^{*}∥{\left(\partial_{I}f\right)}^{*}\left\{-\frac{I^{*}}{∥D^{*}∥}{\hat{D}}_{11}-\frac{I^{*}}{2p^{*}}\hat{p}\right\}$

In (3.5d), the function *f* contains *p*, *ϕ* and *I* as implicit arguments. As reflected in the table, the dependence on *p* and *ϕ* contributes zeroth-order terms in these variables to the linearization.

In (3.5a), (3.5b), *τ* also depends on *p*, *ϕ* and *I*, and the terms involving *τ* are differentiated; hence new issues arise in linearizing them. For example, by the chain rule,

∂j[τ cos⁡(2ψ)]=cos⁡(2ψ){∂pτ∂jp+∂ϕτ∂jϕ+∂Iτ[2dp/ρ∗∂j∥D∥−d∥D∥p3/ρ∗∂jp]}−2τ sin⁡(2ψ)∂jψ.

Since ψ*=0, the full linearization of, say, the first term here equals ${\left(\partial_{p}\tau \right)}^{*}\partial_{j}\hat{p}$, a term given in the table, plus lower-order terms

$${\left(\partial_{j}p\right)}^{*}\left\{{\left(\partial_{pp}\tau \right)}^{*}\hat{p}+{\left(\partial_{\phi p}\tau \right)}^{*}\hat{\phi }+{\left(\partial_{Ip}\tau \right)}^{*}\left[-\frac{I^{*}}{∥D^{*}∥}{\hat{D}}_{11}-\frac{I^{*}}{2p^{*}}\hat{p}\right]\right\}.$$

All of these terms, as well as numerous other analogous terms in the full linearization of (3.5a) that are not of maximal order, have been dropped in (3.10a)–(3.10e).

Putting all the pieces together, we obtain the linearization^8^ of the system (3.5a)–(3.5e)

3.10*a* $$\begin{array}{rl} & \rho_{*}\phi^{*}d_{t}^{*}{\hat{u}}_{1}+A(-\partial_{11}{\hat{u}}_{1}+\partial_{12}{\hat{u}}_{2})+{\left(\partial_{\phi }\tau \right)}^{*}\partial_{1}\hat{\phi }+(1+B)\partial_{1}\hat{p}+2\tau^{*}\partial_{2}\hat{\psi }=0, \end{array}$$

3.10*b* $$\begin{array}{rl} & \rho_{*}\phi^{*}d_{t}^{*}{\hat{u}}_{2}+A(\partial_{12}{\hat{u}}_{1}-\partial_{22}{\hat{u}}_{2})-{\left(\partial_{\phi }\tau \right)}^{*}\partial_{2}\hat{\phi }+(1-B)\partial_{2}\hat{p}+2\tau^{*}\partial_{1}\hat{\psi }=0, \end{array}$$

3.10*c* $$\begin{array}{rl} & d_{t}^{*}\hat{\phi }+\phi^{*}(\partial_{1}{\hat{u}}_{1}+\partial_{2}{\hat{u}}_{2})=0, \end{array}$$

3.10*d* $$\begin{array}{rl} & (1+C)\partial_{1}{\hat{u}}_{1}+(1-C)\partial_{2}{\hat{u}}_{2}-2∥D^{*}∥{\left(\partial_{\phi }f\right)}^{*}\hat{\phi }+\Gamma \hat{p}=0 \end{array}$$

3.10*e* $$\begin{array}{rl} \text{and}\; & \partial_{2}{\hat{u}}_{1}+\partial_{1}{\hat{u}}_{2}+4∥D^{*}∥\hat{\psi }=0, \end{array}$$

where

3.11 $$\begin{array}{rl} d_{t}^{*} & =\partial_{t}+u_{1}^{*}\partial_{1}+u_{2}^{*}\partial_{2},\;A=\frac{I^{*}}{2∥D^{*}∥}{\left(\partial_{I}\tau \right)}^{*},\;B={\left(\partial_{p}\tau \right)}^{*}-\frac{I^{*}}{2p^{*}}{\left(\partial_{I}\tau \right)}^{*} \end{array}$$

and

3.12 $$\begin{array}{rl} C & =f^{*}+I^{*}{\left(\partial_{I}f\right)}^{*}\;\text{and}\;\Gamma =-2∥D^{*}∥\left({\left(\partial_{p}f\right)}^{*}-\frac{I^{*}}{2p^{*}}{\left(\partial_{I}f\right)}^{*}\right). \end{array}$$

Observe that, by hypothesis (2.19), *B*=*C*, a fact that we use in (4.3) and below.

## 4. Proofs, part II: calculation of growth rates

### (a) The eigenvalue problem

We now look for exponential solutions of (3.10a)–(3.10e),

4.1 $$\hat{U}(x,t)=e^{i\langle \xi ,x\rangle +\lambda t}\tilde{U},$$

where $\tilde{U}=({\tilde{u}}_{1},{\tilde{u}}_{2},\tilde{\phi },\tilde{p},\tilde{\psi })$ is a 5-vector of scalars, ***ξ***=(*ξ*_1_,*ξ*_2_) is a vector wavenumber, 〈,〉 indicates the inner product and *λ* is the growth rate. The function (4.1) is a solution of (3.10a)–(3.10e) iff $\lambda ,\tilde{U}$ satisfies the generalized eigenvalue problem

4.2 $$S\tilde{U}=-(\lambda +i\langle u^{*},\xi \rangle )E\tilde{U},$$

where $u^{*}=(u_{1}^{*},u_{2}^{*})$,

4.3 $$S=\left[\begin{array}{ccccc} A\xi_{1}^{2} & -A\xi_{1}\xi_{2} & i{\left(\partial_{\phi }\tau \right)}^{*}\xi_{1} & (1+B)i\xi_{1} & 2i\tau^{*}\xi_{2}\\ -A\xi_{1}\xi_{2} & A\xi_{2}^{2} & -i{\left(\partial_{\phi }\tau \right)}^{*}\xi_{2} & (1-B)i\xi_{2} & 2i\tau^{*}\xi_{1}\\ i\phi^{*}\xi_{1} & i\phi^{*}\xi_{2} & 0 & 0 & 0\\ (1+B)i\xi_{1} & (1-B)i\xi_{2} & -2∥D^{*}∥{\left(\partial_{\phi }f\right)}^{*} & \Gamma & 0\\ i\xi_{2} & i\xi_{1} & 0 & 0 & 4∥D^{*}∥ \end{array}\right]$$

and

4.4 $$E=\left[\begin{array}{ccccc} \rho_{*}\phi^{*} & & & & \\ & \rho_{*}\phi^{*} & & & \\ & & 1 & & \\ & & & 0 & \\ & & & & 0 \end{array}\right].$$

On the right side of (4.2), the modified eigenvalue parameter is *λ*+*i*〈u*,***ξ***〉 because

$$d_{t}^{*}\;e^{i\langle \xi ,x\rangle +\lambda t}=(\lambda +i\langle u^{*},\xi \rangle )\;e^{i\langle \xi ,x\rangle +\lambda t}.$$

Equation (4.2) is a *generalized* eigenvalue problem because ***E***, the matrix of coefficients of time-derivative terms, is not invertible. To extract an ordinary eigenvalue problem, we decompose ***S*** into blocks

4.5 $$S=\left[\begin{array}{cc} S_{11} & S_{12}\\ S_{21} & S_{22} \end{array}\right],$$

where

4.6 $$S_{11}=\left[\begin{array}{ccc} A\xi_{1}^{2} & -A\xi_{1}\xi_{2} & i{\left(\partial_{\phi }\tau \right)}^{*}\xi_{1}\\ -A\xi_{1}\xi_{2} & A\xi_{2}^{2} & -i{\left(\partial_{\phi }\tau \right)}^{*}\xi_{2}\\ i\phi^{*}\xi_{1} & i\phi^{*}\xi_{2} & 0 \end{array}\right]$$

and ***S***_12_, ***S***_21_ and ***S***_22_ fill out the rest of the matrix. Defining ${\tilde{U}}_{1}=({\tilde{u}}_{1},{\tilde{u}}_{2},\tilde{\phi })$ and ${\tilde{U}}_{2}=(\tilde{p},\tilde{\psi })$, we rewrite (4.2) as

4.7 $$\left[\begin{array}{cc} S_{11} & S_{12}\\ S_{21} & S_{22} \end{array}\right]\left[\begin{array}{c} {\tilde{U}}_{1}\\ {\tilde{U}}_{2} \end{array}\right]=-(\lambda +i\langle u^{*},\xi \rangle )E\left[\begin{array}{c} {\tilde{U}}_{1}\\ {\tilde{U}}_{2} \end{array}\right].$$

The zero entries in the last two rows of ***E*** mean that $S_{21}{\tilde{U}}_{1}+S_{22}{\tilde{U}}_{2}=0$ so we can solve for

4.8 $${\tilde{U}}_{2}=-S_{22}^{-1}S_{21}{\tilde{U}}_{1}.$$

Substitution of ${\tilde{U}}_{2}$ into (4.7) then reduces this problem^9^ to the ordinary 3×3 eigenvalue problem,

4.9 $$E_{11}^{-1}[S_{11}-S_{12}S_{22}^{-1}S_{21}]{\tilde{U}}_{1}=-(\lambda +i\langle u^{*},\xi \rangle ){\tilde{U}}_{1},$$

where ***E***_11_ is the 3×3 block in the upper left of ***E***.

We decompose the 3×3 matrix in (4.9) into smaller blocks,

4.10 $$\left[\begin{array}{cc} \frac{\left(M+N\right)}{\rho_{*}\phi^{*}} & \frac{iV}{\rho_{*}\phi^{*}}\\ i\phi^{*}\xi^{T} & 0 \end{array}\right]{\tilde{U}}_{1}=-(\lambda +i\langle u^{*},\xi \rangle ){\tilde{U}}_{1},$$

where we calculate

4.11 $$M=A\left[\begin{array}{cc} \xi_{1}^{2} & -\xi_{1}\xi_{2}\\ -\xi_{1}\xi_{2} & \xi_{2}^{2} \end{array}\right]$$

as the contribution of ***S***_11_,

4.12 $$N=\left[\begin{array}{cc} \frac{{\left(1+B\right)}^{2}}{\Gamma }\xi_{1}^{2}+\frac{\tau^{*}}{2∥D^{*}∥}\xi_{2}^{2} & \frac{\left(1-B^{2}\right)}{\Gamma }\xi_{1}\xi_{2}+\frac{\tau^{*}}{2∥D^{*}∥}\xi_{1}\xi_{2}\\ \frac{\left(1-B^{2}\right)}{\Gamma }\xi_{1}\xi_{2}+\frac{\tau^{*}}{2∥D^{*}∥}\xi_{1}\xi_{2} & \frac{{\left(1-B\right)}^{2}}{\Gamma }\xi_{2}^{2}+\frac{\tau^{*}}{2∥D^{*}∥}\xi_{1}^{2} \end{array}\right]$$

as the contribution of $-S_{12}S_{22}^{-1}S_{21}$, which is symmetric, and

4.13 $$V=\left[\begin{array}{c} \left({\left(\partial_{\phi }\tau \right)}^{*}+\frac{2(1+B)∥D^{*}∥{\left(\partial_{\phi }f\right)}^{*}}{\Gamma }\right)\xi_{1}\\ \left(-{\left(\partial_{\phi }\tau \right)}^{*}+\frac{2(1-B)∥D^{*}∥{\left(\partial_{\phi }f\right)}^{*}}{\Gamma }\right)\xi_{2} \end{array}\right].$$

### (b) Estimation of the eigenvalues

We claim that the growth-rate eigenvalues (4.10) satisfy

$$\max_{j=1,2,3}\;\underset{\xi \in R^{2}}{\sup}\;ℜ\lambda_{j}(\xi )<\infty .$$

By compactness, it suffices to prove that

4.14 $$\max_{j=1,2,3}\;\underset{|\xi |\to \infty }{lim sup}\;ℜ\lambda_{j}(\xi )<\infty .$$

Since only the real parts of eigenvalues matter, we may drop the term *i*〈u*,***ξ***〉 in (4.10) and verify (4.14) for the eigenvalue problem^10^

4.15 $$P\tilde{U}=-\lambda \tilde{U},$$

where we write

4.16 $$P=\left[\begin{array}{cc} \frac{\left(M+N\right)}{\rho_{*}\phi^{*}} & \frac{iV}{\rho_{*}\phi^{*}}\\ i\phi^{*}\xi^{T} & 0 \end{array}\right]$$

for the matrix in (4.10) and we shorten the notation by dropping the subscript 1 on $\tilde{U}$. For large ***ξ***, it is instructive to use perturbation theory to compare the eigenvalues (4.15) with the eigenvalues $P_{0}\tilde{U}=-\Lambda \tilde{U}$, where

4.17 $$P_{0}={\left(\rho_{*}\phi^{*}\right)}^{-1}\left[\begin{array}{cc} M+N & 0\\ 0 & 0 \end{array}\right].$$

**Lemma 4.1**

Provided ***ξ***≠0, the 2×2 matrix ***M***+***N*** is positive definite.

**Proof.**

Since ***M*** and ***N*** are symmetric, it suffices to show that the trace and determinant of ***M***+***N*** are positive. According to (2.20), *A*>0 and *Γ*>0, from which it follows immediately that tr(***M***+***N***)>0.

Regarding the determinant, for any 2×2 matrices

4.18 det(M+N)=detM+detN+χ(M,N),

where

4.19 $$\chi (M,N)=M_{22}N_{11}+M_{11}N_{22}-M_{12}N_{21}-M_{21}N_{12}$$

accounts for the cross terms. For the specific matrices (4.11) and (4.12),  detM=0,

4.20*a* $$\begin{array}{rl} \det N & =\frac{2\tau^{*}}{4\Gamma ∥D^{*}∥}[{\left(1+B\right)}^{2}\xi_{1}^{4}-2(1-B^{2})\xi_{1}^{2}\xi_{2}^{2}+{\left(1-B\right)}^{2}\xi_{2}^{4}] \end{array}$$

4.20*b* $$\begin{array}{rl} & =\frac{2\tau^{*}}{4\Gamma ∥D^{*}∥}{\left[(1+B)\xi_{1}^{2}-(1-B)\xi_{2}^{2}\right]}^{2}\geq 0 \end{array}$$

and

4.21 $$\chi (M,N)=\frac{\tau^{*}}{2∥D^{*}∥}\xi_{1}^{4}+\left(\frac{4}{\Gamma }+\frac{\tau^{*}}{∥D^{*}∥}\right)\xi_{1}^{2}\xi_{2}^{2}+\frac{\tau^{*}}{2∥D^{*}∥}\xi_{2}^{4}>0.$$

This proves the lemma. ■

**Remark 4.2**

It is noteworthy that detN>0 except for the two directions

4.22 $$\frac{\xi_{1}}{\xi_{2}}=\pm \sqrt{\frac{1-B}{1+B}}.$$

Effectively, this calculation rederives the result of Pitman & Schaeffer [12] that the equations of CSSM, even without *I*-dependence, are well-posed for all directions except possibly those defined by (4.22).

It follows from lemma 4.1 that $P_{0}\tilde{U}=-\Lambda \tilde{U}$ has two eigenvalues, say *Λ*_1_,*Λ*_2_, where *Λ*_1_,*Λ*_2_<0 and is homogeneous of degree 2 in ***ξ***. Since ***P*** is an O(|ξ|)-perturbation of ***P***_0_, two of the growth-rate eigenvalues of (4.15) satisfy

$$\lambda_{j}=\Lambda_{j}+O(|\xi |),\;j=1,2,$$

both of which are negative in the limit |ξ|→∞; i.e. they are bounded above by zero in this limit. The third growth rate is given by

$$\lambda_{3}=-\frac{\det P}{\lambda_{1}\lambda_{2}}=-\frac{\det P}{\Lambda_{1}\Lambda_{2}}+O(|\xi {|}^{-1}).$$

The first term on the extreme right is the ratio of two quartics, the denominator being non-zero, so it is bounded, and the perturbation decays at infinity. This verifies (4.14) for all three eigenvalues derived from (3.10a) to (3.10e).

It remains to consider the effect of the lower-order terms that were neglected in (3.10a)–(3.10e). Inclusion of these terms would lead, after a calculation as above, to an eigenvalue problem (4.15) for a perturbed matrix

$$\left[\begin{array}{cc} \frac{M+N}{\rho_{*}\phi^{*}}+O(\xi ) & \frac{iV}{\rho_{*}\phi^{*}}+O(1)\\ i\phi^{*}\xi^{T}+O(1) & O(1) \end{array}\right].$$

As above, two of the eigenvalues of this matrix are negative and $O(|\xi {|}^{2})$, and invoking the determinant shows that the third is bounded. This verifies (4.14) for eigenvalues of the full linearization of (3.5a)–(3.5e) and hence shows that the system is linearly well-posed. It is, therefore, expected that numerical solutions of the full two-dimensional nonlinear transient equations will not exhibit the exponential blow-up of perturbations or the dependence on grid resolution seen in the incompressible *μ*(*I*) equations [9].

## 5. Chute flow in compressible *I*-dependent rheology

Let us recall the steady-uniform ‘Bagnold’ solution [4,31] to the (incompressible) *μ*(*I*)-rheology [4,5] for chute flow. Assuming that *μ*(*I*) is given by (2.13), these solutions exist for inclination angles *ζ* between the maximum and minimum angles $\zeta_{2}=\tan^{-1}(\mu_{2})$ and $\zeta_{1}=\tan^{-1}(\mu_{1})$, respectively. Letting *Oxz* be a Cartesian coordinate system, with the *x*-axis pointing downslope and the *z*-axis being the upward pointing normal, the Bagnold solution is

5.1 $$p_{\mathrm{bag}}=\rho_{⋆}\phi_{⋆}g(h-z)\cos\zeta ,\;\phi_{\mathrm{bag}}=\phi_{⋆}\;\mathrm{and}\;u_{\mathrm{bag}}=\frac{2I_{\zeta }}{3d}\sqrt{\phi_{⋆}g\cos\zeta }(h^{3/2}-{\left(h-z\right)}^{3/2}),$$

where *h* is the flow depth, *ϕ*_⋆_ is a constant solids volume fraction and *u* is the downslope velocity. At a fixed inclination, the inertial number is equal to the positive constant

5.2 $$I_{\zeta }=I_{0}\left(\frac{\tan\zeta -\mu_{1}}{\mu_{2}-\tan\zeta }\right).$$

There is strong experimental and discrete element method (DEM) evidence [4,5,31,32] for both the lithostatic pressure distribution and the three-halves power in the dependence of the velocity on the thickness in (5.1). Fortunately, the CIDR solution is close to this, as we now show.

Assume that all variables depend only on *z* and only the *x*-component of the velocity is non-zero, say *u*=*u*(*z*). Motivated by Bagnold flow, the scalings

5.3 $$p=(\rho_{⋆}gh\cos\zeta )\overset{ˇ}{p},\;z=h\overset{ˇ}{z}\;\mathrm{and}\;u=\left(\frac{I_{\zeta }}{d}\sqrt{g\cos\zeta }h^{3/2}\right)\overset{ˇ}{u}$$

are used to non-dimensionalize the variables and the following ordinary differential equations (ODEs) are derived for $\overset{ˇ}{p}$ and $\overset{ˇ}{u}$ below.

**Lemma 5.1**

*Under the above assumptions, the non-dimensional pressure $\overset{ˇ}{p}(z)$ and the non-dimensional downslope velocity $\overset{ˇ}{u}(z)$ predicted by the CIDR rheology satisfy*

5.4 $$\frac{d\overset{ˇ}{p}}{d\overset{ˇ}{z}}=-\phi$$

*and*

5.5 $$\frac{d\overset{ˇ}{u}}{d\overset{ˇ}{z}}=\sqrt{\overset{ˇ}{p}},$$

*where the pressure is zero at the free surface $\overset{ˇ}{p}(1)=0,$ the velocity is zero at the base $\overset{ˇ}{u}(0)=0$ and for the function *C*(*ϕ*) proposed in (2.30) and (2.31) the solids fraction $\phi =\phi (\overset{ˇ}{p})$ is*

5.6 $$\phi =\phi_{\min}+\Delta \phi \frac{2\chi \overset{ˇ}{p}}{1+2\chi \overset{ˇ}{p}},\;\text{with}\;\chi =\frac{bh}{\beta (I_{\zeta })d}.$$

**Figure 4.**
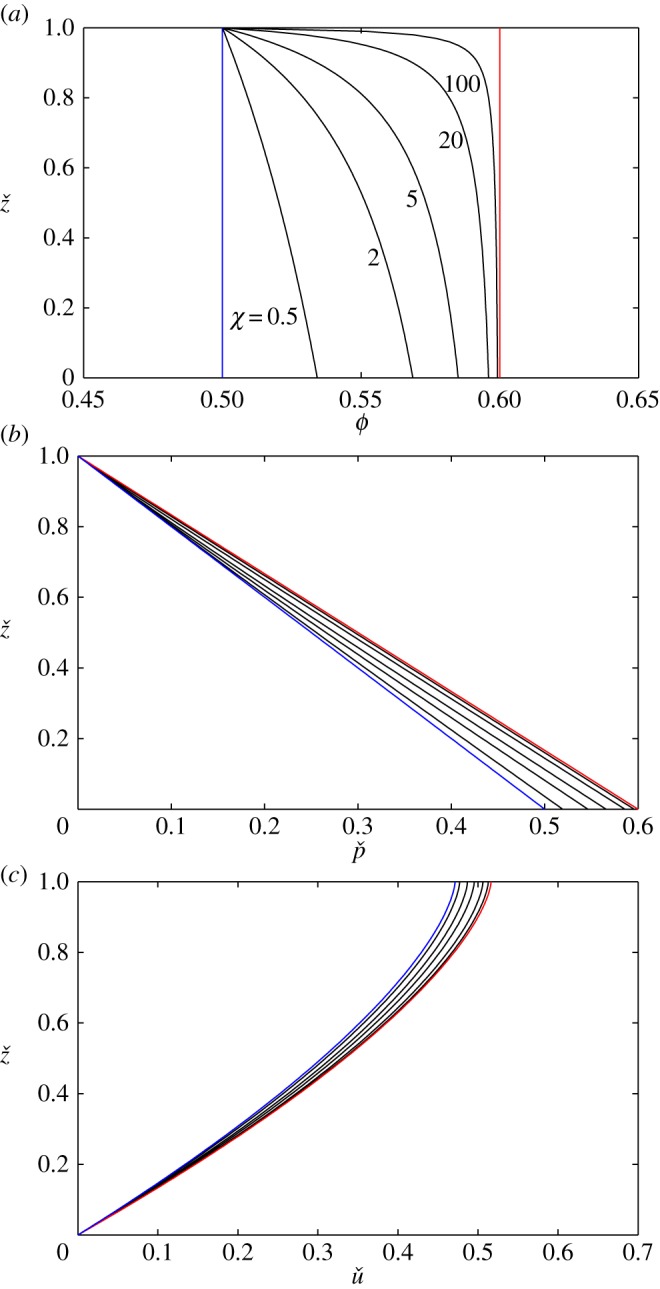
Solutions for (*a*) the solids volume fraction *ϕ*, (*b*) the non-dimensional pressure $\overset{ˇ}{p}$ and (*c*) the non-dimensional velocity $\overset{ˇ}{u}$ as a function of the non-dimensional thickness $\overset{ˇ}{z}$ for five different values of the non-dimensional parameter *χ*=0.5, 2, 5, 20, 100 (black lines). The red line corresponds to χ→∞ and Bagnold flow with *ϕ*_⋆_=*ϕ*_max_, while the blue line corresponds to *χ*→0 and Bagnold flow with *ϕ*_⋆_=*ϕ*_min_. In all cases, *ϕ*_min_=0.5 and *ϕ*_max_=0.6.

**Figure 5.**
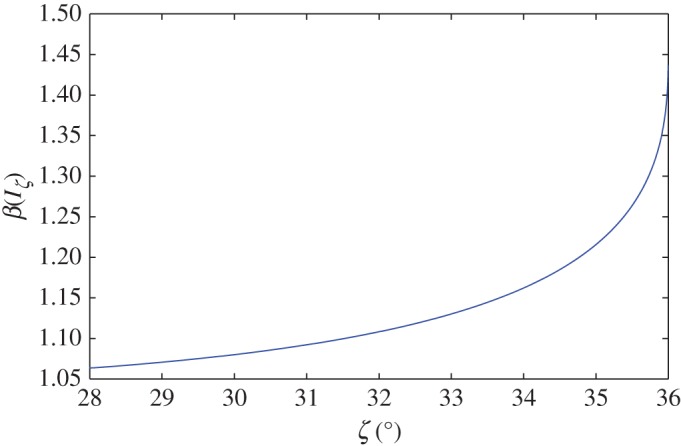
The function *β*(*I*_*ζ*_) as a function of the inclination angle *ζ* for the case *ζ*_1_=28^°^, *ζ*_2_=36^°^ and *I*_0_=0.279. (Online version in colour.)

Steady uniform solutions to the CIDR model are shown in figure 4 for *χ*=0.5, 2, 5, 20, 100 and with *ϕ*_max_=0.6 and *ϕ*_min_=0.5. The nature of the solution is controlled by the non-dimensional parameter *χ*, which is larger for thicker flows. This parameter is also inversely proportional to *β*(*I*_*ζ*_), which by (5.2) and (2.27) is a weakly increasing function of the inclination angle as shown in figure 5. For large *χ*, the concentration graphed in figure 4*a* is close to *ϕ*_max_ over a significant proportion of the flow depth, with a narrow boundary layer near the free surface, where the concentration decreases to *ϕ*_min_. In the limit as χ→∞, the solution tends to the red line in figure 4*a*, which corresponds to Bagnold flow with *ϕ*_⋆_=*ϕ*_max_. As *χ* is decreased, the surface boundary layer becomes thicker and the flow becomes progressively more dilute, tending to the blue line in figure 4*a* as *χ*→0, which corresponds to Bagnold flow with *ϕ*_⋆_=*ϕ*_min_. In figure 4*b*,*c*, the non-dimensional pressure and the velocity are shown, which are also bounded between the red and blue lines corresponding to Bagnold flow with *ϕ*_⋆_ equal to *ϕ*_max_ and *ϕ*_min_, respectively. As well as the profiles being bounded by two non-dimensional Bagnold solutions, the steady-uniform CIDR solutions also have exactly the same scaling properties (5.3), on the flow density *ρ*_⋆_, gravity *g*, the flow depth *h*, the chute inclination *ζ*, the particle diameter *d* and the inertial number *I*_*ζ*_, as the Bagnold solution (5.1). The steady-uniform CIDR solutions are, therefore, very closely related to the classical Bagnold solution and are almost indistinguishable for large *χ*.

**Proof of the lemma.**

The form of the assumed solution implies that *D*_*xx*_=*D*_*zz*_=0 and *D*_*xz*_=(1/2) d*u*/d*z*>0; in particular, div ***u***=0. It follows from the alignment condition (2.8) that *σ*_*xx*_=*σ*_*zz*_=−*p* and *σ*_*xz*_=*τ*, where *τ*=||***τ***||. Thus, the downslope and normal momentum balances (2.2) reduce to

5.7 $$\frac{d\tau }{dz}+\rho_{*}\phi g\sin\zeta =0$$

and

5.8 $$\frac{dp}{dz}+\rho_{*}\phi g\cos\zeta =0.$$

We may apply the chain rule to (5.7) and (5.8) to derive dτ/dp=tan⁡ζ, and, since tractions at the surface vanish,

5.9 τ=ptan⁡ζ.

On the other hand, since div ***u***=0, the flow rule (2.18) implies *f*(*p*,*ϕ*,*I*)=0, and, given the ansatz (2.24b), we conclude that

5.10 $$p=\frac{1}{2}\beta (I)C(\phi ).$$

From (5.10), we have *p*/*C*(*ϕ*)=*β*(*I*)/2, so by (2.24b) and (2.25)

5.11 $$\tau =\left[\alpha (I)-\frac{\beta (I)}{2}\right]p=\mu (I)p.$$

Comparing (5.9) with (5.11), it follows that μ(I)=tan⁡ζ, and hence the inertial number *I* is equal to the constant *I*_*ζ*_ everywhere. The value of *I*_*ζ*_ is given by the formula (5.2), which is derived by inverting (2.13) with $\mu (I_{\zeta })=\tan\zeta$. Since the inertial number is constant and *C*(*ϕ*) satisfies (2.30), equation (5.10) can be inverted and then non-dimensionalized, using the scalings (5.3), to give the solids volume fraction $\phi =\phi (\overset{ˇ}{p})$ in (5.6). Using the same scalings (5.3), the normal momentum component (5.8) yields the ODE (5.4), which can be solved for the pressure $\overset{ˇ}{p}$ by integrating down from the free surface using (5.6). The concentration $\phi (\overset{ˇ}{p})$ then follows from (5.6). From the definition of the inertial number (2.12) and that 2∥***D***∥=|d*u*/d*z*|, the downslope velocity satisfies the ODE

5.12 $$\frac{du}{dz}=\frac{I_{\zeta }}{d}\sqrt{\frac{p}{\rho_{*}}}$$

and the scalings (5.3) then give the ODE (5.5), which, since $\overset{ˇ}{p}$ is known, can be integrated upwards from the no-slip condition $\overset{ˇ}{u}(0)=0$ at the base. ■

## 6. Conclusion and discussion

In this paper, we have analysed a generalization of the *μ*(*I*)-rheology that allows for changes in the granular solids volume fraction. The equations of motion, the CIDR model, are found to be linearly well-posed when the constitutive laws satisfy certain criteria. We indicate (in §2e) how the specific *I*-dependence of the *μ*(*I*)-rheology can be fitted into the theory, and we have shown that the inclusion of compressibility removes the ill-posedness of the incompressible rheology [9].

CIDR equations for steady, uniform flow down an inclined chute are solved in §5, where we observe that the solution is comparable to the classical Bagnold solution under conditions similar to the many experimental and DEM results [4,5,31,32] for the velocity profile in steady chute flows. Crucially, the equations are *well-posed at all inertial numbers*, which corresponds to the full range of inclination angles for steady chute flow, in contrast with the limited range of angles in which the incompressible theory is well-posed [9]. At the same time, the new theory captures the dilatant behaviour of over-consolidated granular material as the structure of the equations is motivated by CSSM.

Incidentally, the chute flow calculation relates to Forterre & Pouliquen [33], who modify incompressible *μ*(*I*)-rheology by postulating a constitutive law in which *ϕ* is explicitly specified as a function of the inertial number, say *Φ*(*I*). In the CIDR model, there is no constitutive law relating these two variables; nevertheless, dependence of *ϕ* on *I* is implicit in the solution. It is also quite possible that a more subtle dependence of volume fraction on inertial number will be needed to reproduce some phenomena reported recently in experiments and numerical simulations, while retaining the property of well-posedness.

Our primary viewpoint in this paper has been to regard the CIDR model as modifying *μ*(*I*)-rheology with compressibility. However, it is equally valid to regard CIDR as modifying CSSM with rate dependence. To recapitulate, our result shows well-posed equations result from such modification provided that the yield locus and flow rule satisfy (2.19) and (2.20). Unlike in the incompressible *μ*(*I*)-rheology and the rate-independent CSSM equations, the CIDR equations are linearly well-posed for all deformations and for perturbations in all directions in Fourier space.

An important next step will be to specify constitutive laws, satisfying the general conditions for linear well-posedness of this paper, and formulated to accurately match the available experimental results and discrete numerical simulations for granular flows such as two-dimensional steady flow in a Couette geometry and time-dependent chute flow. Then the CIDR model and well-posedness result can be tested with fully two-dimensional nonlinear transient numerical computations of these flows.

## Figures and Tables

**Figure 6.**
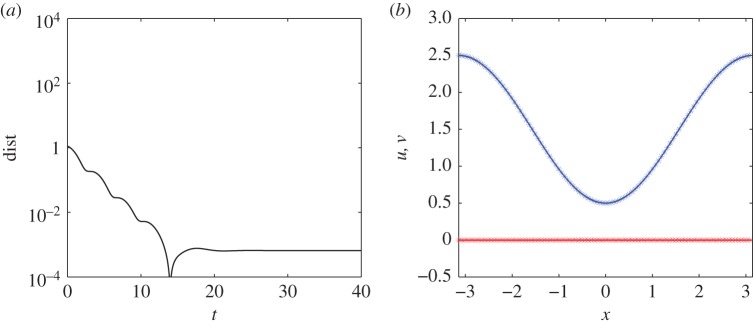
Numerical solutions of (B 4) with the distance from the asymptotic solution (B 15) in (*a*) and the fields at *t*=100 in (*b*). Here *a*=1.5, *ε*=0.01 and the discretization is *Δx*=2*π*/100 and *Δt*=1×10^−3^.

**Figure 7.**
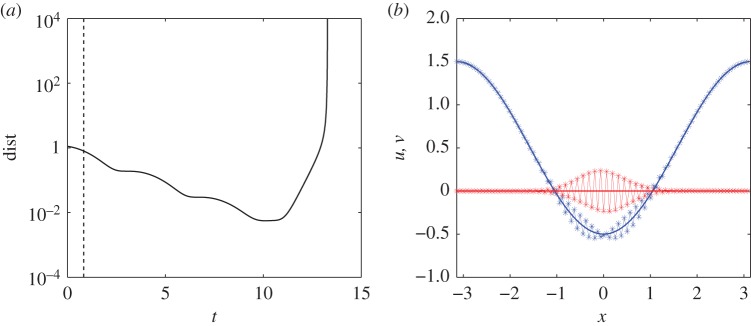
Numerical solutions of (B 4) with the distance from the asymptotic solution (B 15) in (*a*) and the fields at *t*=12.5 in (*b*). Here *a*=0.5, *ε*=0.01 and the discretization is *Δx*=2*π*/100 and *Δt*=1×10^−3^. The vertical dashed line in (*a*) is the first time that *u*=0.

**Figure 8.**
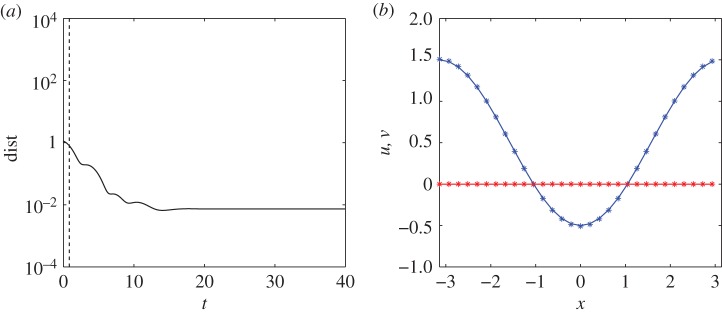
Numerical solutions of (B 4) with the distance from the asymptotic solution (B 15) in (*a*) and the fields at *t*=100 in (*b*). Here *a*=0.5, *ε*=0.01 and the discretization is *Δx*=2*π*/30 and *Δt*=1×10^−3^. The vertical dashed line is the first time that *u*=0.

## Footnotes

1 Thus, (2.5) contains the implicit assumption that material is actually deforming. Otherwise, (2.5) must be replaced by inequality, ∥***τ***∥≤*μp*, and the governing equations are underdetermined unless further relations, such as those of elasticity, are included.

2 Note that, for incompressible flow, the full strain-rate tensor (∂_*j*_*u*_*i*_+∂_*i*_*u*_*j*_)/2 and the deviatoric strain-rate tensor are equal as the second term on the right in (2.7) vanishes.

3 If *Y* and *f* are independent of *I*, then (2.19) leads to the CSSM flow rule (.2) derived from normality.

4 Note that *P* in fact depends only on div ***u***,∥***D***∥ and *ϕ*.

5 Note that we are assuming isotropy, which may be a questionable assumption at such low densities.

6 Hence ***σ*** has eigenvalues −*p*±∥***τ***∥. Note that −*p*−∥***τ***∥ is the major stress eigenvalue—although this eigenvalue is the smaller algebraically, it is the larger in absolute value.

7 It is also true that $(\partial_{1}u_{2}+\partial_{2}u_{1})\sin2\psi \leq 0$, and if cos⁡2ψ were to vanish, we would need to use this inequality to guarantee that *k*<0. However, this issue will not arise in the analysis below.

8 These equations are maximal order except that in (3.10a) and (3.10b) the term $d_{t}^{*}{\hat{u}}_{j}$ retains first-order spatial derivatives even though these equations also contain second-order derivatives of ${\hat{u}}_{j}$.

9 In other words, we are performing on the symbol level the reduction that we performed on the operator level in §2d.

10 Do not forget the minus sign in this equation—the growth rates are *negative* eigenvalues of ***P***.

## Appendix A. Ideas from critical-state soil mechanics

**(a) Constitutive equations**

CSSM is an ingeniously constructed version of plasticity that includes compressibility but reduces to a singular perturbation of Coulomb material, which is incompressible, in an appropriate limit. In two-dimensional CSSM, flow is described by the usual six variables, *ϕ*, ***u*** and ***σ***. Since flow is compressible, the solids fraction *ϕ* remains as a genuine variable. The governing equations consist of the conservation laws (2.1), (2.2) plus three constitutive laws. One of the constitutive equations is the alignment condition (2.8), with no changes required. The second constitutive equation, like (2.5), specifies the norm of the deviatoric stress,

A 1 Yield condition: ∥τ∥=Y(p,ϕ),

but as indicated the function *Y* depends on the solids fraction *ϕ* as well as on the mean stress *p*. The final constitutive relation, the flow rule, relates expansion and contraction of material to the slope of the yield surface,

A 2 $$\text{Flow rule:}\;\text{div}\;u=2\frac{\partial Y}{\partial p}(p,\phi )\;∥D∥.$$

We refer to Jackson [1] for a derivation of (A 2) from the normality condition of plasticity.

By way of example, a simple, physically acceptable, yield locus is given by

A 3 $$Y(p,\phi )=2\mu p-\frac{p^{2}}{C(\phi )},$$

where *μ* is a coefficient of friction, as in (2.5), and *C*(*ϕ*) is an increasing function of the solids fraction of the form (2.30). For such a yield condition, it follows from the proof of lemma 2.5, restricted to the case where *μ*(*I*) is independent of *I*, that equations (2.1), (2.2), (A 1), (A 2), (2.8) reduce to the Coulomb model in the limit *Δϕ*→0.

**(b) Consequences of the flow rule**

The behaviour discussed in this subsection occurs under fairly general hypotheses—see Jackson [1]. However, to explain the theory with a minimum of technicalities, we confine the discussion to the specific yield condition (A 3).

The phrase *critical state*, from which CSSM derives its name, refers to a state *p*,*ϕ* such that

A 4 $$\frac{\partial Y}{\partial p}(p,\phi )=0.$$

For the example yield condition (A 3), condition (A 4) means that

A 5 $$2\left(\mu -\frac{p}{C(\phi )}\right)=0.$$

Rewriting the yield condition as *Y* (*p*,*τ*)=[2*μ*−*p*/*C*(*ϕ*)]*p* and invoking (A 5), we deduce that at a critical state

A 6 ∥τ∥=μp.

The set where (A 6) is satisfied is called the critical state line. Thus, *along the critical state line, the stress satisfies the Coulomb yield condition.*

According to the flow rule (A 2), at a critical state, deformation is not accompanied by any change in *ϕ*. Let us examine behaviour away from the critical state line. Suppose that, for example, initially the (uniform) state of material is at yield at point A in figure 2*b*. At this point, ∂*Y*/∂*p*<0, so according to the flow rule div ***u***<0; i.e. material compactifies and becomes stronger, so *τ* must increase for deformation to continue. Indeed, the stress will continue to increase until a critical state on a larger yield surface is reached, as suggested in figure 2*b* by the *ϕ*_3_-yield surface. Moreover, if *Δϕ* in (2.30) is small, a very slight increase in *ϕ* is sufficient to accommodate this evolution. That is, we expect stress to be quickly driven from point A to a critical state on a larger yield surface where the Coulomb yield condition (A 6) is satisfied.

Conversely, at point B in figure 2*b*, ∂*Y*/∂*p*>0, so under deformation div ***u***>0; i.e. material expands and becomes weaker. It is natural to imagine that the stress is driven to a critical state on a smaller yield surface, as suggested by the arrow in the figure. This would indeed be the case *if material deformed uniformly*, but this assumption is unrealistic for stresses above the critical state line, *τ*>*μp*. For such stresses, because material expands under deformation and therefore weakens, instability often causes localized deformation—if deformation near one point happens to be slightly larger than elsewhere, the associated expansion lowers the yield condition more near this point, and subsequent deformation tends to concentrate near this point.

## Appendix B. A primer on ill-posed partial differential equations

The following appendix gives a self-contained, elementary summary of key issues regarding ill-posed PDEs. A much more detailed treatment can be found in [6].

**(a) Testing for ill-posedness**

The initial value problem for a PDE is called *well-posed* in the sense of Hadamard if for general initial data a solution (1) exists, (2) is unique and (3) varies continuously under perturbations of the initial conditions^11^ (see also Pinchover & Rubinstein [34]). If one or more of these criteria is not satisfied then the problem is called *ill-posed*. A classic example of an ill-posed problem is the backward heat equation

B 1 $$\partial_{t}u=-\partial_{xx}u.$$

In appendix Bb, we show that condition (1) fails; here we show that condition (3) also fails. Taking the Fourier transform reveals that the equation admits solutions

B 2 $$u_{\xi }(x,t)=\sin(\xi x)\;e^{\xi^{2}t},$$

for any ξ∈R. Consider the scaled solutions |*ξ*|^−*p*^*u*_*ξ*_(*x*,*t*), where *p*>0, as perturbations of the trivial solution *u*(*x*,*t*)≡0. The initial conditions of the scaled solution—i.e. $|\xi {|}^{-p}\sin(\xi x)$—tend to zero in the sup norm as ξ→∞; indeed, if *p*>*k* these initial conditions tend to zero in the $C^{k}$ norm. On the other hand, for any *t*>0, the norm

B 3 $$\underset{x\in R}{\sup}|\xi {|}^{-p}u_{\xi }(x,t)$$

tends to infinity in this limit. Thus, an arbitrarily small perturbation of initial conditions for (B 1) can lead to an arbitrarily large solution in an arbitrarily short time.

For more general PDEs, there is a test for ill-posedness based on Fourier analysis of the *linearization* of the equations. The process is summarized as:

(i) linearize the equations about a base-state solution;
(ii) freeze the coefficients at some point (***x****,*t**);
(iii) look for solutions with exponential dependence *e*^*i*〈***ξ***,***x***〉+*λ*(***ξ***)*t*^.

We shall say the original PDE is linearly ill-posed (with respect to the base-state solution at the given point) if

$$\underset{|\xi |\to \infty }{lim sup}\lambda (\xi )=+\infty .$$

For most examples, if a PDE is linearly ill-posed, it is ill-posed in the sense of Hadamard. (But see Kreiss [35] for exceptional examples.)

An equation is called *linearly well-posed* with respect to a given base solution if the growth rate is bounded from above for all points (***x****,*t**). Linear well-posedness does not imply well-posedness in the sense of Hadamard. For example, it is trivially verified that the Navier–Stokes equations are linearly well-posed, but a major effort is required to show that, even just for a finite time, they are well-posed in the sense of Hadamard, and it is not known whether they are well-posed for all time.

We illustrate the above test on the following made-up nonlinear system that has some similarity to the PDEs analysed in this paper:

B 4 $$\begin{array}{rl} \partial_{t}u & =\partial_{x}v\\ \text{and}\;\partial_{t}v & =\varepsilon u\partial_{xx}v+\partial_{x}u-v-\sin(x). \end{array}\}$$

The linearized equations with frozen coefficients are

B 5 $$\begin{array}{rl} \partial_{t}\hat{u} & =\partial_{x}\hat{v}\\ \text{and}\;\partial_{t}\hat{v} & =\varepsilon [u^{*}\partial_{xx}\hat{v}+{\left(\partial_{xx}v\right)}^{*}\hat{u}]+\partial_{x}\hat{u}-\hat{v}, \end{array}\}$$

where *u**,*v** is the base-state solution evaluated at the point (*x**,*t**) and $\hat{u},\hat{v}$ are the perturbations solutions

B 6 $$\hat{U}=\left[\begin{array}{c} \hat{u}\\ \hat{v} \end{array}\right]=e^{i\xi x+\lambda t}\tilde{U},$$

where $\tilde{U}\in R^{2}$ satisfies the eigenvalue problem

B 7 $$\left[\begin{array}{cc} 0 & i\xi \\ i\xi +\varepsilon \partial_{xx}v^{*} & -\varepsilon u^{*}\xi^{2}-1 \end{array}\right]\tilde{U}=\lambda \tilde{U}.$$

The eigenvalues of (B 7) could be easily calculated exactly but, provided that *u**≠0, they can be estimated more easily from their asymptotic behaviour as |ξ|→∞,

B 8 $$\lambda_{1}=-\varepsilon u^{*}\xi^{2}+O(\xi )\;\text{and}\;\lambda_{2}=\frac{\det S(\xi )}{\lambda_{1}}=-\frac{1}{\varepsilon u^{*}}+O(\xi^{-1}),$$

where *S*(***ξ***) is the 2×2 matrix in (B 7). If *u**>0, then the eigenvalues satisfy

B 9 $$\max_{j=1,2}\underset{|\xi |\to \infty }{lim sup}\;ℜ\lambda_{j}(\xi )<\infty ,$$

so (B 4) is linearly well-posed. On the other hand, if *u**<0, then *λ*_1_ is unbounded, so (B 5) is ill-posed.

Note that, in analysing linear ill-posedness of (B 10), we consider the *full* linearization of the equations, i.e. (B 5). One might be tempted to discard terms with lower-order derivatives in the expectation that the growth of exponential solutions as |ξ|→∞ ought to be dominated by the highest-order derivatives in the equation. However, the counter-example

$$\partial_{t}u=\partial_{xxx}u-\partial_{xx}u$$

shows that this expectation is not valid, in general.

Nevertheless, for this example we may in fact analyse exponential solutions of (B 5) by first considering the *maximal-order* linearized equations

B 10 $$\begin{array}{rl} \partial_{t}\hat{u} & =\partial_{x}\hat{v}\\ \text{and}\;\partial_{t}\hat{v} & =\varepsilon u^{*}\partial_{xx}\hat{v}+\partial_{x}\hat{u}. \end{array}\}$$

In each of the above equations, only terms of maximal order are retained, i.e. the terms ${\left(\partial_{xx}v\right)}^{*}\hat{u}$ and $\hat{v}$ have been dropped from the second equation because it contains the higher-order terms $\partial_{x}\hat{u}$ and $u^{*}\partial_{xx}\hat{v}$, respectively. The growth rate of exponential solutions of (B 10) satisfy the same estimates (B 8), and the neglected lower-order terms do not change the leading-order behaviour. Often calculations may be simplified by studying the maximal-order linearization as an intermediate step.

**(b) Consequences of ill-posedness**

**(i) Restrictions on the existence of solutions**

In order for an ill-posed initial value problem to have a solution, usually the initial conditions must satisfy an extreme smoothness requirement, stronger than is physically acceptable in most applications. It may be difficult to demonstrate this behaviour, in general, but, for the backwards heat equation, we illustrate the behaviour with the following.

Proposition B.1 If *ε*≠0, the initial value problem for (B1) with initial data $u(x,0)=\varepsilon |\sin x{|}^{p}$ has no solution for any positive time interval unless the power *p* is an even non-negative integer.^12^

Proof. Suppose the 2*π*-periodic function *f*(*x*) has Fourier series $f(x)∼\sum c_{n}\;e^{inx}.$ If equation (B 1) with initial condition *u*(*x*,0)=*f*(*x*) has a continuous solution for 0≤*t*<*η*, it has the Fourier series representation

$$u(x,t)=\sum_{n=-\infty }^{\infty }c_{n}\;e^{inx+n^{2}t}.$$

Moreover, for 0≤*t*<*η*

B 11 $$\sum_{n=-\infty }^{\infty }\;e^{2n^{2}t}|c_{n}{|}^{2}=\frac{1}{2\pi }\int_{-\pi }^{\pi }|u(x,t){|}^{2}\;dx<\infty .$$

Now if the Fourier coefficients of a function *f*(*x*) satisfy $\sum n^{2k}|c_{n}{|}^{2}<\infty ,$ then the derivatives (*d*/*dx*)^*j*^*f*(*x*) are square integrable for *j*=0,1,…,*k*. But, provided *p* is not an even non-negative integer, the proposed initial data $|\sin x{|}^{p}$ has singular behaviour near *x*=0. Specifically, ${\left(d/dx\right)}^{k}|\sin x{|}^{p}$ is square integrable only if *k*<*p*+1/2. It follows for the Fourier coefficients of $|\sin x{|}^{p}$ that, if *k*>*p*+1/2,

$$\sum_{n=-\infty }^{\infty }n^{2k}|c_{n}{|}^{2}=\infty .$$

This inequality is incompatible with (B 11), so the initial value problem cannot be solved on any positive time interval. ■

**(ii) Grid-dependent computations**

The attempt to solve an ill-posed PDE numerically produces unreliable, grid-dependent, results. Such behaviour has been observed in various physical problems [7–10] where the formulation was based on an ill-posed system of equations. However, in complicated problems like these, usually computational resources are stretched to the limit, meaning behaviour under grid refinement cannot be readily probed. Let us illustrate grid dependence on a much less demanding problem, the toy problem (B 4) above.

If *ε*=0 and with initial conditions

B 12 $$u(x,0)=a\;\mathrm{and}\;v(x,0)=\frac{-\sin(x)}{2},$$

the (linear) equations (B 4) have the exact solution

B 13 $$\begin{array}{rl} u(x,t) & =\frac{e^{-t/2}}{2}\left[\cos\left(x+\frac{\sqrt{3}t}{2}\right)+\cos\left(x-\frac{\sqrt{3}t}{2}\right)\right]+a-\cos x\\ \text{and}\;v(x,t) & =\frac{e^{-t/2}}{2}\left[\cos\left(x+\frac{\sqrt{3}t}{2}+\frac{\pi }{6}\right)-\cos\left(x-\frac{\sqrt{3}t}{2}-\frac{\pi }{6}\right)\right]. \end{array}\}$$

The large-time limit of these solutions,

B 14 u(x,∞)=a−cos⁡xandv(x,∞)=0,

is also a steady-state solution of the nonlinear system (with *ε*>0). If *a*>1, then u(x,∞)>0, and the calculations above suggest that the equations will be linearly well-posed. However, if *a*<1, then u(x,∞) dips below zero over an interval, which suggests that the equations will be ill-posed.

Figure 6, where *a*=1.5, and figure 7, where *a*=0.5, confirm these expectations. They show numerical solutions using a central-space forward-time explicit scheme on the periodic domain *x*∈[−*π*,*π*] with a spatial resolution of *Δx*=2*π*/100. Each figure has two panels, one showing the temporal evolution of the distance from the asymptotic solution,

B 15 $$\text{Dist}=\max\left\{\sqrt{|u-u(x,\infty ){|}^{2}+|v-v(x,\infty ){|}^{2}}\right\},$$

and the other plotting the two variables *u*,*v* at a specific (late) time during the computation. In figure 6, the well-posed case, the numerical solution converges to the predicted steady-state solution (B 14) within numerical accuracy. By contrast, in figure 7, after an initial decay, ill-posedness asserts itself and causes the solution to blow up.

Regarding grid dependence, figure 8 shows another computation in the ill-posed case with a coarser grid, *Δx*=2*π*/30. The solution appears to converge to the steady-state solution, just like in the well-posed case. In other words, the computations on the coarse grid hide the ill-posed character of the underlying PDEs. This highlights that, in order to extract meaningful information from numerical computations, a proper study of grid convergence must be first carried out.

Incidentally, if the grid is made finer than in figures 6 and 7, in the well-posed case *a*>1 the numerical solution converges to the steady-state solution with a smaller numerical error, while in the ill-posed case it blows up *sooner*, as expected.
